# A Patch-Based Method for Repetitive and Transient Event Detection in Fluorescence Imaging

**DOI:** 10.1371/journal.pone.0013190

**Published:** 2010-10-15

**Authors:** Jérôme Boulanger, Alexandre Gidon, Charles Kervran, Jean Salamero

**Affiliations:** 1 Mathematical Imaging, Radon Institute for Computational and Applied Mathematics, Linz, Austria; 2 Cell and Tissue Imaging Core Facility and Nikon Imaging Centre@Institut Curie, Centre National de la Recherche Scientifique-Institut Curie, Paris, France; 3 Molecular Mechanisms of Intracellular Transport, Centre National de la Recherche Scientifique-Institut Curie, Paris, France; 4 Centre Rennes-Bretagne Atlantique, Institut National de Recherche en Informatique et en Automatique, Rennes, France; 5 Unité de Recherche 341 Mathématiques et Informatique Appliquées, Institut National de la Recherche Agronomique, Jouy-en-Josas, France; German Cancer Research Center, Germany

## Abstract

Automatic detection and characterization of molecular behavior in large data sets obtained by fast imaging in advanced light microscopy become key issues to decipher the dynamic architectures and their coordination in the living cell. Automatic quantification of the number of sudden and transient events observed in fluorescence microscopy is discussed in this paper. We propose a calibrated method based on the comparison of image patches expected to distinguish sudden appearing/vanishing fluorescent spots from other motion behaviors such as lateral movements. We analyze the performances of two statistical control procedures and compare the proposed approach to a frame difference approach using the same controls on a benchmark of synthetic image sequences. We have then selected a molecular model related to membrane trafficking and considered real image sequences obtained in cells stably expressing an endocytic-recycling trans-membrane protein, the Langerin-YFP, for validation. With this model, we targeted the efficient detection of fast and transient local fluorescence concentration arising in image sequences from a data base provided by two different microscopy modalities, wide field (WF) video microscopy using maximum intensity projection along the axial direction and total internal reflection fluorescence microscopy. Finally, the proposed detection method is briefly used to statistically explore the effect of several perturbations on the rate of transient events detected on the pilot biological model.

## Introduction

In this study we focus on image sequences showing M10 cells stably expressing Langerin-YFP. This cell line was first introduced in [1]. Langerin is a constitutively endocytosed and recycled trans-membrane receptor, [2], [3]. To get deeper insights in the recycling pathway and dynamics of this molecule, an array of microscopy assays has been developed, including Fast 4D microscopy associated or not with F-techniques. Among diverse molecular behaviors reported by preliminary video microscopy studies on this molecular model, membrane concentration of fluorescently tagged proteins gives rise to sudden spot appearance and disappearance. Total Internal Reflection Fluorescence (TIRF) microscopy demonstrated that these transient fluorescence concentrations can occur close to or at the plasma membrane, suggesting that they may correspond to membrane sites specialized in endocytosis or exocytosis of the Langerin. However fast 4D or TIRF microscopy showed that hundreds of such events may occur every minute at different places within one single cell.

The analysis of endo/exocytosis process in TIRF microscopy and in electronic microscopy has been investigated in [4] and in [5]. The authors proposed to analyze the spatial distribution of the vesicles. In particular, in [4], a semi-automatic detection method based on morphological operators is used to locate the vesicles fusion in space and time. Furthermore, a point process modeling is proposed in order to characterize some properties of the endo/exocytosis process such as the presence of clusters. Several traditional image processing approaches and motion detection techniques can be considered also for the automatic detection of fast appearing and vanishing spots in wide-field and TIRF microscopy images. Frame difference thresholding methods are probably the most popular techniques in video analysis [6], [7]. Markov random field modeling is generally recommended to capture local statistics and to represent spatial correlations [8]; nevertheless, one needs to adjust several parameters in practice and iterative energy minimization techniques are required to compute the Maximum A Posteriori estimators [9], [10]. To overcome this difficulty, parameter free approaches have been recently investigated for change detection [11], [12]. All these methodologies including learning-based background subtraction techniques [13], [14] have been considered in our study, but they cannot be applied to process several hundred 2D and 3D image sequences. Actually, it is challenging to distinguish activities due to trafficking and vesicles in motion from activities corresponding to appearances and disappearances of small spots in an image pair. It turns out that learning a reference “background” is not an easy task in our context. Previous methods [15]–[18] do not completely fulfill the specified requirements efficiently. A possible alternative strategy would be to track all the vesicles contained in the cell. However, the performances of existing state-of-the-art methods [19]–[21] are limited by noise, clutter and low temporal resolution, especially in wide-field (WF) microscopy. Typically, tracking approaches would be able to extract fragments of trajectories which would imply a high number of false alarms. In order to quantify accurately, the numbers, the rates and further the half lives of these transient membrane structures, a robust and automatic approach adapted to either 3D or TIRF series of images, is required for which several issues must be addressed. First, the method should be able to distinguish the sudden appearing and vanishing events from movements due to membrane trafficking. Second, the detection method should also adapt to the image quality but should not contain any a priori on the number of events. For instance, it should be able to perform when the number of events per pixel is very low (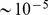). In this sense the representation should be adaptive and sparse [22]. Finally, the method should be automatic enough to be applied with the same parameters to a data-base of image sequences to quantify the influence of applied perturbations.

In this paper, we describe such a method for the estimation of the rate of appearing and vanishing bright spots in image sequences. We tested its accuracy for two quite distinct microscopy modalities, 3D+time WF reduced to 2D+time via maximum intensity projection along the (axial) z direction and TIRF, while for the same biological model submitted to different perturbations. The proposed approach takes into account the photon-limited nature of the images via a variance stabilization transform and in particular for WF microscopy, occurrence of photo-bleaching. We then describe a patch-based approach related to [16] and a simpler frame difference method for the detection of events. Each of them implements two statistical controls and are detailed in the Material and Methods Section. The performance of the two described approaches are tested on synthetic sequences and we demonstrate the ability of our approach to evaluate the influence of drug, physical or molecular perturbation on these events. Finally, we discuss the validity of our approach and its extension to further improvements.

## Materials and Methods

### Biological model preparation and perturbation

M10 melanoma cells have been stably transfected with a plasmid encoding the chimeric protein Langerin-Yellow Fluorescence Protein, and cell lines were selected following the protocol and the criteria already described previously [1] 24h before image acquisition, the M10+Lang-YFP cells are plated on 18mm glass cover-slips and cultivated with RPMI complete medium (foetal calf serum 10%, Geneticin 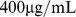) at 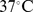, 5% 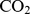. Cover-slips are mounted in a Ludin chamber (a small, stainless steel piece of equipment for 18mm cover-slips that may be utilized as either an open or closed perfusion chamber). For drug treatment, cells are pre-incubated 1h at 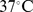, with nocodazole 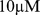 or cytochalasin D 100nM and then visualized during the next hour without washing. For biological perturbation following mutant proteins expression, cells are plated on 18mm glass cover-slips and cultivated with RPMI medium supplemented with 10% FCS, without Geneticin at 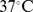, 5% 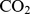, 48 hours before image acquisition. 24 hours before image recording, cells are transiently transfected with a plasmid encoding for the tail-mutant of the Myosin Vb fused to the CFP (pMyoVbtail-GFP was a gift from Jim Goldenring) using 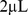 of Plus Reagent (Invitrogen, USA) and 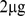 of pMyoVbtail-CFP, completed to 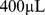 with OptiMEM (Invitrogen, USA) before a 5min incubation at room temperature. 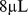 of Lipofectamin LTX (Invitrogen, USA) are then added to this mix and further incubated at room temperature for 30min. These 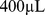 mix is added to 1.6mL of culture medium and incubated 4h at 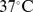. Cells are then washed with complete RPMI medium without Geneticin, and further incubated at 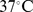, 5% 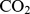, until image recording. For temperature dependency experiments, cells were also pre-incubated at 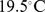 for 1h and sequences were imaged for no more than 1h at 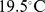.

### Image sequence acquisition

We consider two modalities for image acquisition: wide-field and total internal reflection microscopy.

Wide-field video-microscopy sequences are acquired with a 100× objective (NA = 1.4) on a DMIRB Leica inverted microscope (Leica microsystem, Heidelberg), equipped with a motorized objective piezo (Physik Instrumente, Karlsruhe, Germany) for fast and reproducible Z displacement (the distance between two planes is 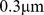). Illumination is performed with a 488nm wavelength generated by a monochromator Polychrome V (Till photonics, GmbH, Munich, Germany). Fluorescent emissions are selected with ET-GFP/mCherry filter (Chroma bloc) and are captured by a QuantEM EMCCD camera (Roper Scientific, Tucson, AZ, USA) driven by Metamorph (Molecular devices, Sunnyvale, CA, USA). The Ludin chamber is placed in a dedicated stage holder and the whole microscope set-up is surrounded by a temperature and 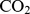 regulated incubating system (LIS, Basel, Switzerland). For all image acquisitions, a 3D stack of 4 planes is acquired every second with 200ms illumination time per frame for a total duration of 120 seconds (1 stack/s). All series correspond to a 2 minutes duration of acquisition. Movies are then generated by compiling 3D stacks over time.

TIRF microscopy sequences are acquired on a Nikon TE 2000 inverted microscope equipped with a TIRF arm (Nikon Instrument, B.V. Amsterdam, Netherlands). We use a high aperture 100× TIRF objective NA = 1.49. For laser illumination, YFP is excited with a 491nm fibered laser diode (laser launch, Roper Scientific). Fluorescent emission is selected with ET-GFP/mCherry filter (Chroma bloc) and are captured on a Quant-EM CCD camera (Roper scientific). The whole microscopic setup is driven by Metamorph software (Molecular devices) and temperature control as well as 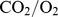 equilibrium is insured using the Cube from Life Imaging Service. Image sequences were exclusively generated in stream mode of acquisition and the exposure time per frame is 100ms for a 1 minute total duration of imaging (about 600 images per series).

### Selection of the molecular model for image analysis at the single cell level

**Figure 1:**
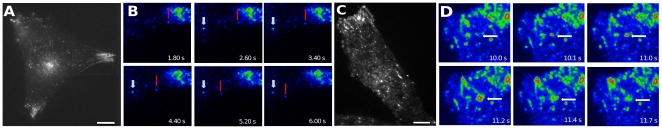
Dynamics of YFP-Langerin in M10 stable cell lines. Maximum Intensity Projection of time lapse video (about 0.8–1 fps) of Langerin-YFP fluorescence (A) and thumbnails temporal series (B) corresponding to the boxed area in (A), recorded in full field illumination fluorescent video-microscopy. Large white arrow shows sudden appearance of fluorescence concentration while thin red arrows identifies lateral movement of a vesicle. The same cell line was imaged using a TIRF microscopy set-up equipped with a very high sensitive EM-CCD camera and a typical Maximum Intensity Projection of a TIRF microscopy video sequence (10fps) is presented in (C). Note that the time regime is different between A and B and that long distance trajectories as detectable in (A) are not present in (C). Thumbnails temporal series (D) of the boxed area in (C) illustrate the time delay between appearance of a fixed spot loaded with Langerin-YFP corresponding to a vesicle entering the evanescent field and docking at a specific site of the cell plasma membrane, its subsequent diffusion and progressive disappearance (D, white arrow).

As a model biological system for single cell studies, we have developed and selected a cell line stably expressing a yellow fluorescent chimeric trans-membrane protein. This choice was a critical issue, since preliminary experiments showed the strongest heterogeneity in both the dynamic behavior and the topological distribution of this protein at the single cell level, when transiently expressed (data not shown). The Langerin, our reporter molecule, known to traffic from the recycling endosomes to the plasma membrane, is naturally expressed by the Langerhans cells of the epidermis [3]. For this reason, our cell line was selected and cloned on the basis of criteria fitting with information available from freshly isolated human Langerhans cells isolated from skin sheets obtained from surgery [23]: 1) the intracellular localization, 2) the level of expression relative to protein concentration and 3) ultra-structural organization. Nevertheless, variation in the fluorescent intensity (within a ratio of 1 to 2), size and shape from cell to cell, corresponding in part to the well known stochasticity of gene expression in single cell studies, were still observed. Moreover, the resulting fluorescence live cell imaging, for instance in WF microscopy, still indicates diverse dynamic behavior for Langerin-YFP, (Figure 1A and Video S1) including lateral and directional movements as well as sudden apparition of concentrated spots. Furthermore, those bright fluorescent spots may be transient or/and vanishing rapidly (Figure 1B), although not systematically. Altogether these factors make the design of a robust approach to automatically record and select particular membrane protein activities a real challenge. In the particular case of intracellular proteins following exocytosis or recycling pathways, a supplementary level of complexity has to be taken into account which is linked to the knowledge of the cellular confinement where those molecules are functional or delivered. In terms of fluorescence imaging it means to be able to distinguish the spatial occurrences of transient events, such as sudden local protein concentration, dissociation or diffusion of one group of those entities. All these dynamical processes cannot be easily followed by a single microscopy technique or by single molecule approaches. We thus aimed to develop a quantitative approach versatile enough to be used either in 3D wide field (Figure 1A) or in TIRF microscopy (Figure 1D & E). Evanescent waves (TIRF) have a number of advantages for recording live cells as compared to wide field microscopy. Among them, it allows to restrain the depth of observation to a very narrow slab, close to the cell plasma membrane. Thus, it constitutes the best microscopy to date for the study of exocytic or endocytic vesicles dynamics. Practically a fluorescently labeled exocytic or recycling vesicle will appear as a sudden and fast event once it enters the evanescent field. As illustrated in Figure 1, both imaging techniques revealed similar repetitive and transient events of fluorescence detection, showing that at least a part of them are indeed occurring at or close to the plasma membrane.

### Event detection

The proposed detection method can be decomposed into three main steps. A first pre-processing step is dedicated to the “normalization” of each image sequence. In the second step we focus on the detection of transient events. Finally, a post-processing step allows to cluster and counts detected events in space and time.

### Image segmentation and normalization

In the case of 3D+time WF microscopy, 3D stacks are first summarized to 2D images via a maximum intensity projection. We consequently loose the three dimensional information but the computational load is reduced while the estimated global rate of event remains the same.

**Figure 2:**
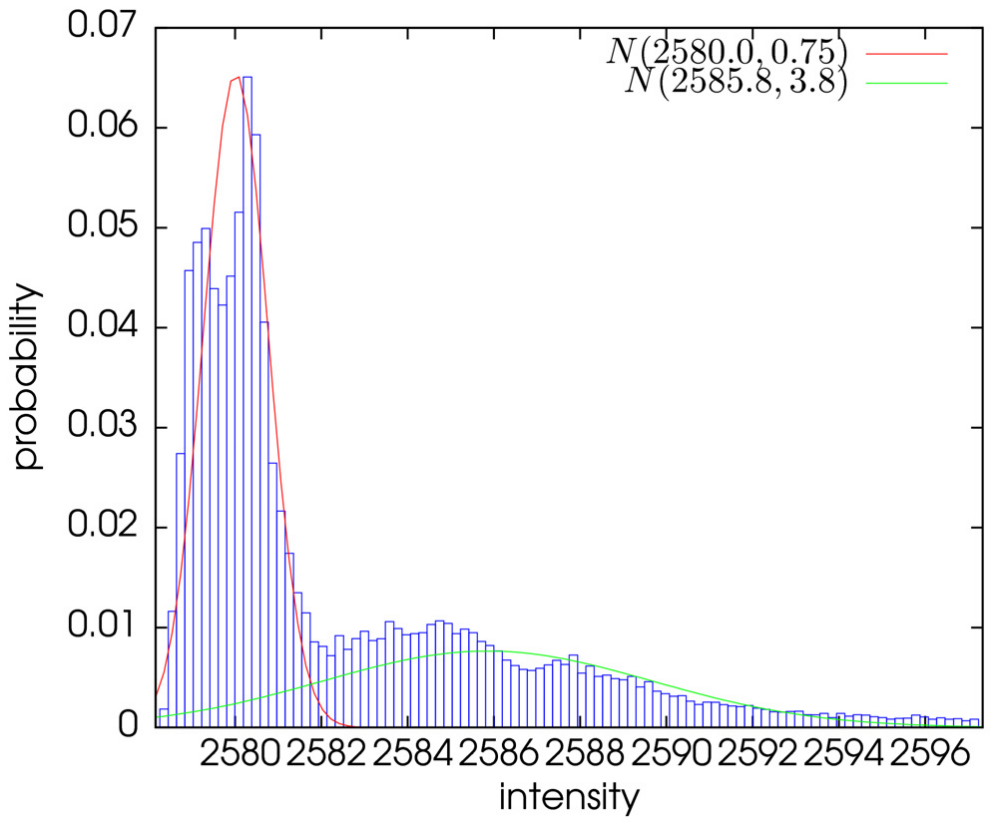
Histogram of the maximum intensity projection (MIP) image of a WF image sequence. The two Gaussian components of the mixture have been estimated using an Expectation-Maximization algorithm.

We delineate roughly the cell boundary using the following image segmentation method. The probability distribution function of the image intensity is modeled by a mixture of two Gaussian distributions 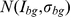 and 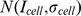 associated respectively to the background and the cell. The parameters of the mixture are estimated using an usual Expectation-Maximization (EM) procedure as shown on Figure 2 [24].

**Figure 3:**
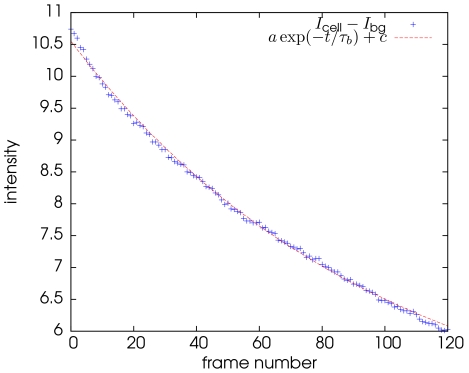
Estimation of the bleaching time constant.

In WF microscopy, photo-bleaching is responsible for large decreases of the signal-to-noise ratio along time. Decreases of 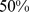 of the average intensity inside the cell are observed along the time sequence. The difference of intensity between two successive 3D frames can be non negligible especially for the first frames of the sequence. In order to correct the effect induced by photo-bleaching, we first make the approximation that the mean of the intensity inside the segmented cell should be constant in time and we consider an exponential model for the decay of the intensities along time:
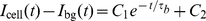
where 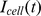 is the mean of the intensities inside the cell and 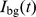 is the mean of the intensities of the background taken from outside the cell. 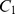 and 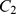 are two constants related to the amplitude and the offset while 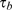 is the time constant of the exponential decay. These three parameters are automatically estimated using a Gauss-Newton minimization (see Figure 3). Note that ideally the offset would be null (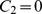). However, in practice we observed that this is not the case due to the possible influence of auto-fluorescence. Finally, we can define the bleaching correction procedure as
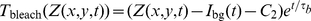
where 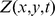 is the measured intensity at pixel 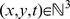. This procedure allows to estimate the intensities as if there would have been no fading and is able to preserve the intensity variations due to vesicles motions for instance. As a result, the noise level increases inherently with time when the procedure is applied.

Moreover, both in WF and TIRF microscopy, limited intensity and additional electronic noises lead to the following affine stochastic Poisson-Gaussian noise model:
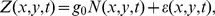
where 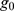 represents the gain of the overall electronic system. The number 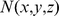 of collected photo-electrons is a random variable assumed to follow a Poisson distribution of parameter 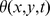. Finally, the contributions of the dark current and of the readout noise are summarized as a Gaussian white noise of mean 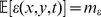 and variance 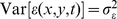.

Consequently, the detection algorithm should then be able to adapt to the variation of the noise variance along with the intensity implied by the presence of shot-noise. Instead, we propose to stabilize the variance of the noise using the Generalized Anscombe Transform defined by [25]:
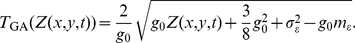
Once this procedure has been applied, the noise variance is theoretically 1 in the entire image sequence. The parameters 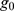, 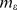 and 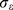 are estimated using the procedure described in [26]. Note that the Anscombe transform has the drawback to lose the linearity of the sensor which induces a deformation of the objects. In particular, given the case of an object whose intensity decreases in time, after stabilization, this object will appear as having a slightly different shape. However, important change in shape or in intensity should lead to a positive response of the detector.

In the case of WF microscopy, one has to combine variance stabilization and bleaching correction. This is done by applying first the variance stabilization and then the bleaching correction: 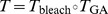 which finally leads to
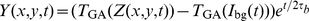
taking into account the effect of the variance stabilization transform on the data. Moreover, the variance of the noise, is also multiplied by the same factor and while the intensities are stable over time, after correction the noise variance increases exponentially as 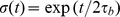. In TIRF microscopy, since only a slice of the cell is imaged, we cannot assume that the intensities are constant and therefore, bleaching correction is impossible. Fortunately, due to the high sensitivity and low sample illumination of this modality, bleaching is often considered negligible and only the generalized Anscombe transform is applied to the acquired data and we set 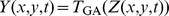.

#### Event detection

The underlying idea of patch-based event detection is to associate for each small image domain of size 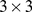 or 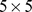 pixels (patch), the best corresponding patch in the next frame. More precisely, we propose to analyze the similarity of matched patches and decide whether they are likely to correspond to the same object. This method is then related to motion estimation and tracking. However, we avoid the problem of association to detect sudden events.

In the proposed method, for each patch around the point of coordinates 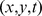, we consider a square search window of size 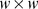 located at the same spatial position 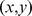 but at the time 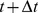. For each point in this search window, we compute the distance (normalized sum of square difference (SSD)) between patches defined as:
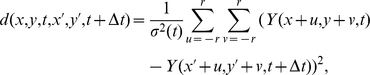
for patches of size 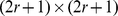 pixels. In order to guaranty the symmetry of the detection procedure, the SSD between the patch located at the location 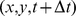 and the patches in the search window of the point 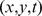 is also computed. Moreover, the noise standard deviation is robustly estimated from the difference between two successive frames.

Finally, we obtain a set 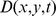 of cardinal 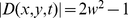 distances corresponding to the number of points in the search window of size 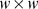 for both directions and removing the extra comparison to the patch at the location 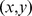 which would be performed twice otherwise. If there is no signal difference between two patches, since 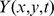 is normally distributed after variance stabilization, the samples of the set 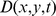 follow then a 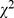 distribution with 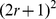 degrees of freedom.

**Figure 4:**
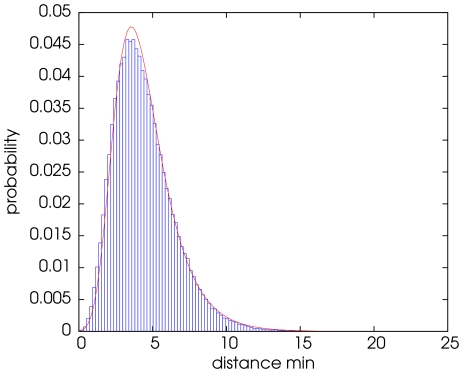
Distribution of the minimum distance between patches. The size of the patches is 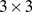 pixels and the sets contains 17 samples corresponding to search windows of size 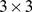. The density of Generalized Extreme Value distribution with the following estimated parameters: 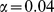, 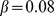 and 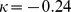 is shown in red.

If one distance in the set 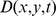 is small enough, we can conclude that a pattern has been matched. This means that either there has been no significant motion or the local tracking of the pattern has been successful. In order to determine if a meaningful change occurred at a location 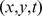, we consider the minimum distance 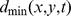 of the set 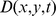. According to the Fisher-Tippett theorem [27], the minimum of a set of well behaved random variables follows the Generalized Extreme Value (GEV) distribution defined as:
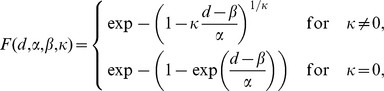
where 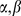 and 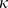 are respectively the width, the location and the shape parameter of the GEV distribution. Moreover, in the case of correlated stationary processes, Berman provided some additionnal conditions on the covariance matrix for the convergence of the maximum (resp. minimum) toward such double exponential density [28]. Figure 4 shows in blue the distribution of the distance between patches of size 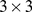 corresponding to a pair of frames extracted from the image sequence shown in Figure 1 A. The parameters of the GEV distribution are estimated using a mixed L-Moments/Maximum Likelihood method [29]. In practice, in order to be more robust to outliers due to motions when estimating the parameters of the GEV distribution on real images, we pre-select the values of 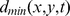 below a 0.999-quantile of the GEV distribution whose parameters have been estimated beforehand on a Monte-Carlo simulation based on some white noise (see Figure 4) for the first image and using the previously estimated parameters otherwise. This procedure allows a certain degree of adaptation but preserves the ability of the proposed method to detect the events of interest. Finally, each video shows the distribution of 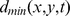 and the fitted GEV distribution.

In the next step, in order to decide if the computed minimum distance 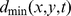 corresponds to an event (hypothesis 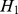) or not (hypothesis 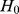), several approaches can be considered. Since the test is performed at each pixel it is then important to consider a multi-hypothesis testing framework such as the Bonferroni-Šidàk correction [30] controlling the Family-Wise Error Rate (FWER) or the Benjamini and Hochberg procedure [31] controlling the False Discovery Rate (FDR). Note that the procedure of Benjamini and Hochberg includes a modification in order to take into account correlated measurements (like in this case).

### Event clustering and counting

In each frame, the “active” detected pixels are grouped into regions using a labelling algorithm. However, if the described method is able to detect satisfyingly fast emergence and fading events, fast movement due to membrane trafficking observed especially in WF microscopy might produce false detections. However, these situations can be discarded by analyzing events lying in the same space-time neighborhood. Thus we propose to apply a Mean-Shift algorithm on the barycenter of the detected “active” regions [32].

### Quantification of the influence of the perturbations

In order to test the significance of the effect on the rate of the events of the described biological perturbations, we can conduct a pairwise analysis of variance (ANOVA) against the reference (wild type). Beforehand, in order to deal with the Poisson statistic induced by the counting process, we propose to stabilize the variance of the number of detected events using the Anscombe transform described earlier.

### Implementation

The proposed algorithm has been implemented in C++. The computation of the SSD and the estimation of the parameters of the GEV distribution have been parallelized in order to reduce computation cost. The parameters of the program are the size of the patch, the size of the search zone, the type of statistical control and the associated 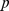-value and the interval 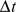 between two frames. Furthermore, the estimation of the parameters of the GEV distribution has two additional parameters: a 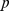-value and the frequency at which the estimation of the parameters is performed which allows to reduce mainly to computational cost by estimating the parameters only a few times along the sequence.

The method we have described is embedded into an automated batch procedure. The parameters are the same for all the image sequences of the data-base. We save the result in a synthetic file summarizing for each sequence: the number of images, the size of images, the gain 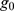 of the camera, the estimated parameters 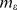 and 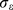 of the electronic noise, the bleaching constant 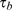, the area of the segmented cell and finally the number of detected appearing or disappearing spots.

## Results

### Simulation study

**Figure 5:**
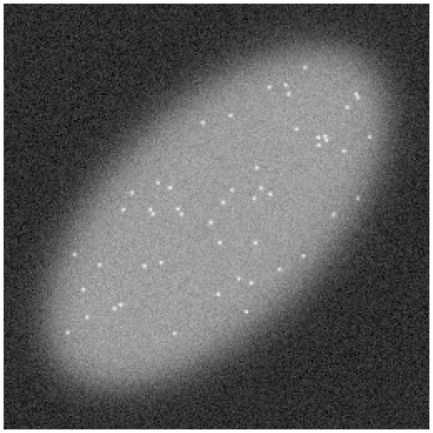
First frame of a typical synthetic image sequence used for testing the detection method.

We propose to test the proposed approach on simulated images sequences in order to evaluate its performances. To obtain realistic images, we have analyzed several real images sequences and tried to obtain the same characteristics. Each generated sequence contains 100 frames of 256
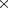
256 pixels. The background intensity is fixed to 2500 intensity levels. A synthetic cell is depicted by an ellipse of 2600 intensity levels. The gain of the sensor is set to 0.1 while the dark current is modeled by a Normal distribution of mean 2000 and standard deviation 3.6 intensity levels. We consider a Gaussian shape for a fixed number of 100 spots and dummy spots moving according to a Gaussian random walk are also generated in addition to the appearing and disappearing spots. The intensity of the spots is added to the background. In order to generate a scale of signal-to-noise ratio, we vary the spot intensity from 20 to 200 intensity levels. Figure 5 shows the first frame a generated sequence with a spot intensity of 100 intensity levels.

For comparison, we have also implemented an alternative approach based on the difference of two successive frames but using the same pre-processing and statistical control (FWER and FDR). This method is related to the recent work presented in [7]. However, if in theory the distribution of the image difference should follow a Normal distribution [6], we experimentally found that a distribution with a heavier tail like the Logistic distribution was able to provide better results. In addition, especially in the case of TIRF microscopy, it appears necessary to overestimate systematically the noise variance in order to reduce the sensitivity to residuals motions. Finally, we can see the frame difference based (FD) approach as a limit of the proposed patch-based (SSD) approach when the patch size and the search window size tend to 1 pixel even although the modeling distributions used for each case remain different.

**Figure 6:**
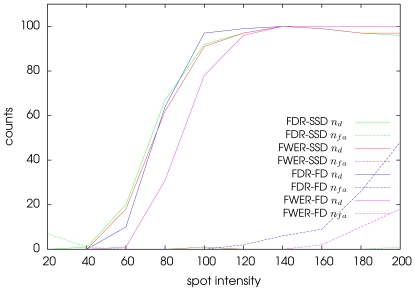
The signal-to-noise ratio influences the number of true detections and false alarms. The number of true detections 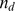 and the number of false alarms 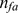 are drawn as a function of the signal-to-noise ratios for the proposed SSD method and for a FD approach [7] using a FDR and a FWER control. The test has been performed on sequences similar to the one shown in figure 5.

We have applied the two methods to a set of 10 sequences. Figure 6 depicts the evolution of the number of false alarms (dashed line) and the positive detections (plain line) for the proposed SSD method and the FD approach using the two proposed control procedures. In both cases, we observe that FDR is more conservative than FWER and provides a higher number of positive detections. In the meanwhile, we can also see that the number of false alarms is not much worst with FDR than with FWER. Finally, we observe surprisingly that the number of false alarms explodes for high signal-to-noise ratios with the FD approach. This can be interpreted as the effect of the dummy spots whom movements start to be distinguishable from noise. This experiment illustrates the robustness of the proposed SSD approach to motions due to traffic, especially present in WF microscopy. Finally, when the number of spots in motion decreases, the two methods tend to provide similar results.

### Validation on acquired data

**Figure 7:**
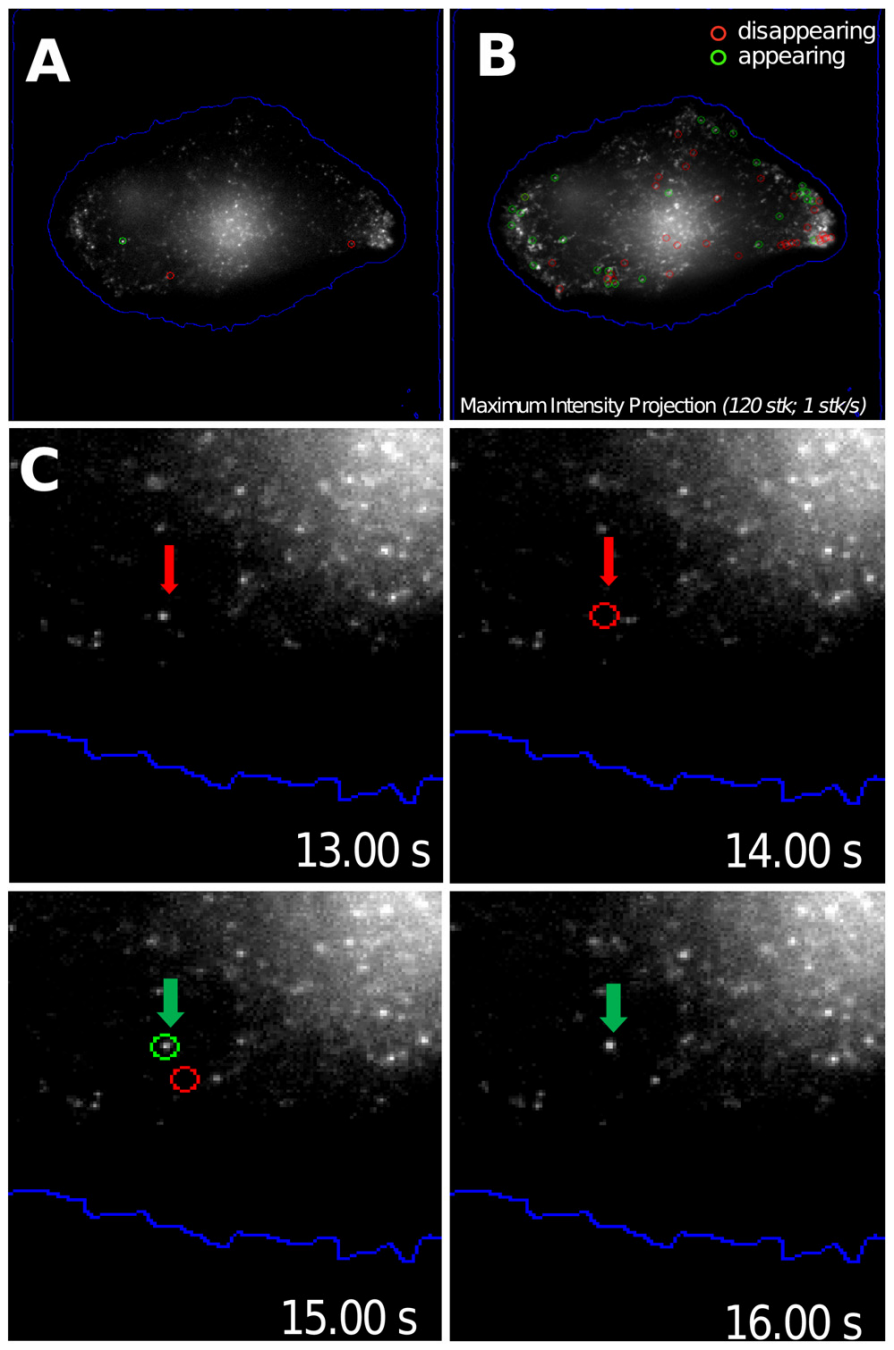
Validation of the proposed method through biological, drugs and physical perturbation. A detection map in a single image corresponding to a 2D projection of a 3D stack (A) and a Maximum Intensity Projection of Video S2 acquired in the WF mode (B) are represented. Green circles identify appearing spot and red circles disappearing spots. The cell contour is delineated in blue and obtained by automatic segmentation. Thumbnails sequence in (C) shows automatic detection of suddenly disappearing structure (red circles and arrows) and appearing spots (green circles and arrows) at the frame rate of one 3D stack per second.

The accuracy assessment of the detection counts and of the quality of the events detected were performed directly on the images. This was done in the biological context described earlier in this paper. Figure 7 illustrates the detection of appearing and vanishing fluorescent Langerin spots on single cell sequences. Figure 7 A represents one 2D projection of one single 3D stack of a sequence acquired in WF microscopy (Video S2). Automatic cell segmentation is superimposed on the image (blue line). From the whole sequence, a Maximum Intensity Projection is performed (Figure 7B). Appearing (green circles) and vanishing (red circles) structures are detected and thumbnails (Figure 7C) corresponding to local detections within the indicated temporal window (from t = 13s to t = 16s) are presented.

In order to evaluate the robustness and the accuracy of our approach in the case of real image sequences, we have applied the proposed method to a data-base of acquired image sequences. This data-base contains respectively five and three different conditions applied to the Langerin biological model in WF and TIRF microscopy. This model was chosen for its sensitivity to a number of experimental perturbations with effects on the dynamics of the events ranging from moderate to profound [23]. Langerin is a trans-membrane protein routed through the tubulovesicular network of endosomal recycling compartment (ERC), thus trafficking along cytoskeleton elements. We first recorded series of sequences acquired in WF microscopy, on cells treated with cytochalasin D or nocodazole, two drugs known to affect the polymerization of actin microfilaments and microtubules, respectively. As a low temperature blocks or reduces membrane trafficking, cells were imaged at 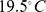. As a recycling membrane receptor, Langerin obeys to a series of cytosolic machineries regulating or driving different transport steps. Among them, Langerin dynamics is under the strict control of Rab11A [2], a small GTPase involved in the overall plasticity of functional domains of the ERC. As considered in a more integrative view, none of the unique molecular species within a cell works as an isolated entity. The molecular motor Myosin Vb is a known Rab11A interacting protein [33]. A deleted mutants of the motor domain of this protein, consequently affects the dynamics of recycling endosomes [33] and of their content, such as the reporter protein followed in this study. The proposed approach was thus tested also in Langerin positive cells over-expressing the Myosin Vb Tail mutant tagged with-CFP. Sequence series were then acquired either in 3D WF or in TIRF microscopy, following recording conditions as explained in experimental procedures.

Among a large number of sequences (between 46 and 12 different sequences per experimental condition), Videos S3 and S4, represent examples of processing on single cells for nocodazole or cytochalasin D in WF microscopy and Videos S5 and S6, examples on control (without treatment) or MyoVb Tail expressing cells in TIRF microscopy, respectively.

**Figure 8:**
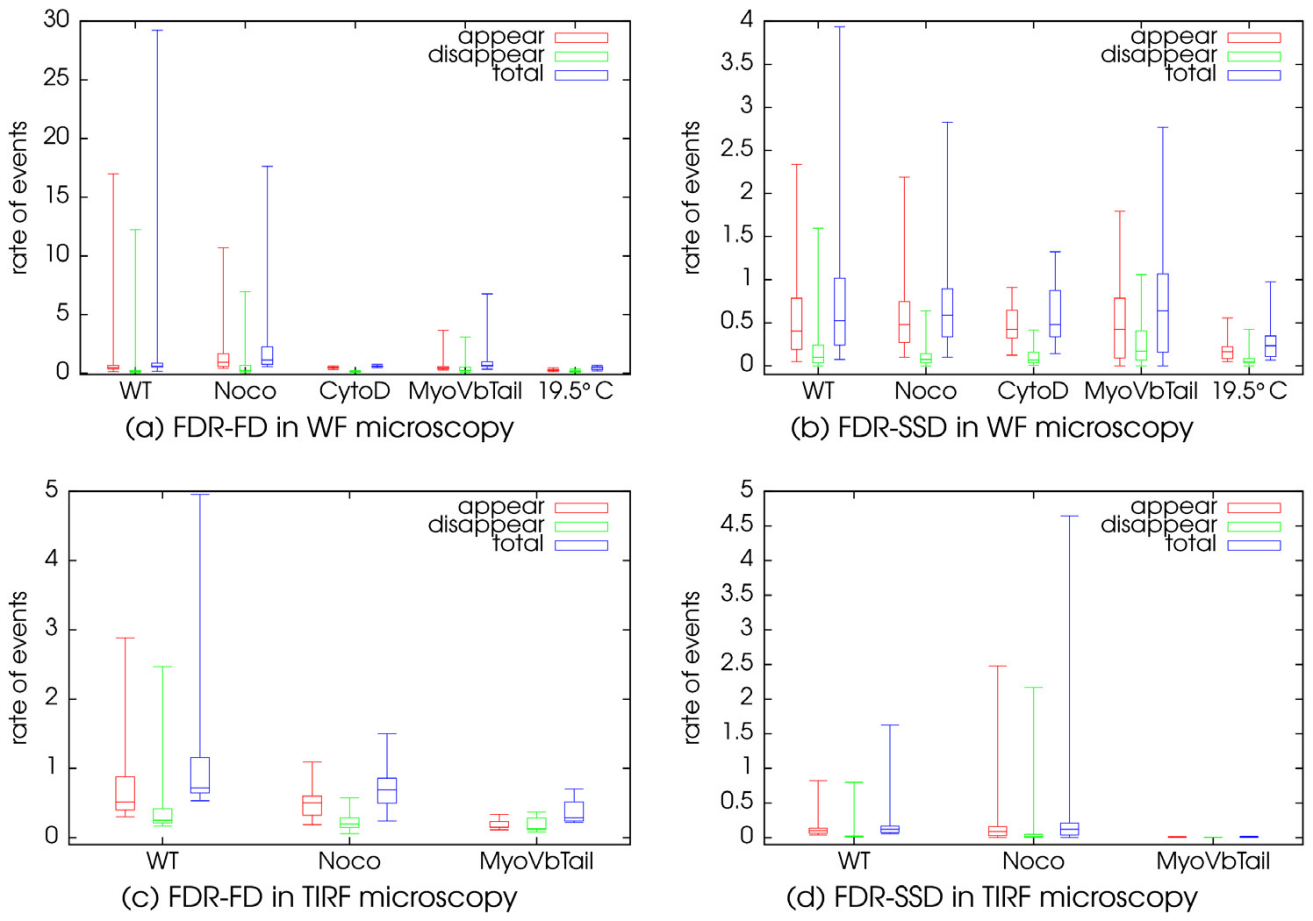
Distribution of the rate of detected events at the single cell level. The FD approach and the proposed SSD approach have been applied in the same conditions of bleaching compensation and variance stabilization.

The combination of described statistical controls with variance stabilization and bleaching correction allows one to apply the proposed method without tuning its parameters for each image sequence. Nevertheless, the distinction between image sequence acquired in WF and TIRF microscopy remains necessary at this stage. The processing of the 187 image sequences acquired in WF microscopy took 52 min for the SSD approach and 27 min for the FD approach using in both cases a FDR control while the processing of the 62 image sequence acquired in TIRF microscopy took respectively 1 h 8 min and 33 min using the same statistical control. This processing has been carried out on a 2GHz quad-core desktop computer. The SSD approach used a 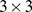 patches and 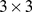 search windows and the parameters of the GEV distribution were robustly estimated on the data. The result of the event detection for both approaches (FD and SSD) and both modalities (WF and TIRF) are illustrated on Figure 8 showing the rate of events for each sequence defined as the number of events divided by the number of images in the sequence (exposure times are constant within a given modality). This representation allows us to evaluate the distribution of the rate of events and to perform a visual side-by-side comparison of the results provided by the proposed SSD approach and the FD method. Furthermore, in order to quantify the effect of the different perturbations on the rate of events, a pairwise analysis of the variance was performed. A summary of the results obtained with both approaches is shown in Table 1. The comparison of these figures should be conducted with care since the perturbation may also affect membrane trafficking.

**Table 1:** Analysis of variance.

Modality	Method	Perturbation	n	Mean	Std	P
WF	FDR-DF	wild type	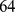	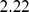	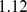	-
		nocodazol	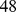	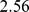	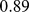	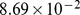
		cytochalasin D	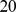	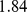	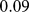	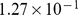
		Myosin Vb tail	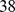	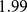	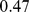	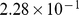
		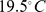	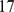	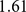	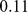	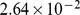
	FDR-SSD	wild type	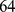	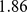	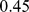	-
		nocodazol	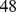	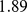	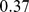	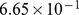
		cytochalasin D	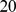	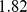	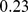	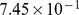
		Myosin Vb tail	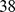	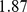	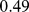	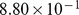
		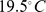	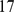	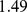	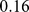	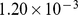
TIRF	FDR-FD	wild type	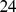	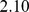	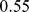	-
		nocodazol	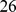	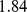	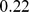	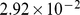
		Myosin Vb tail	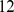	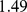	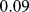	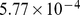
	FDR-SSD	wild type	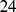	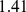	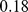	-
		nocodazol	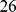	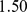	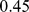	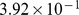
		Myosin Vb tail	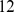	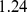	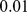	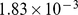

Results of the analysis of variance of the rate of total number of event detected (see Figure 8) by the two detection methods and the two image modalities (WF and TIRF). Note that the Anscombe variance stabilization transform has been applied beforehand in order to improve homoscedasticity.

### Preliminary analysis

An interesting number of confirmations and new conclusions can be drawn from our automatic method. While microtubules partly drive the displacements of ERC membranes and organize the steady state geometry of their localization within the cells, depolymerization after nocodazole treatment significantly increases the frequency of fast appearing and disappearing spots in WF (in both detection techniques), indicating that this fluorescent behavior most probably correspond to the local gathering of multiple entities of Langerin molecules. Depending on its expression level, MyoVb Tail mutant leads to the relative but significant decrease in the number of events detected by the SSD approach, more evidently in the TIRF microscopy modality consistent with the fact that Myosin Vb acts primarily as a molecular motor, driving vesicular movement. Despite the fact that bleaching estimation and camera gain are taken into account, it will be hazardous at this stage to cross-compare quantitative data obtained with the proposed approach in WF and TIRF microscopy, because exposure times (200ms in WF and 100ms in TIRF microscopy) and the exposure mode (Time-lapse and Stream) were not comparable. However, an important issue was to assess the adequacy of the robustness for both technologies.

Practically, acquiring with TIRF microscopy a fluorescently labeled exocytic or recycling vesicle will appear as a sudden event, once it enters the evanescent field. On the contrary, a structure, such as an endocytic vesicle should disappear more or less fast, once it enters the interior of the cell. Our method is particularly suitable for the automatic recording of such behavior. Here only 3 different experimental conditions were examined for detection in TIRF microscopy (Figure 8 C and D): untreated cells (WT; n = 24), Cells treated with nocodazole (noco; n = 26) and cells overexpressing the MyoVbTail mutant (MyoVb Tail; n = 12). A reproducible and significant decrease in both appearing and disappearing spots in the latter condition, demonstrated that MyoVb is active for the access of Langerin to the plasma membrane that is in the recycling step of this protein. While it was somehow expected, this constitutes a first direct result of the proposed methodology.

In TIRF images of nocodazole treated cells, we have detected a slightly reduced mean number of events as compared to untreated cells, giving a reverse picture of their relative tendency in WF microscopy. While these results from the automatic detection have to be taken with care and need further investigations, the qualitative survey of the experimentalist on the image data of all detected spots, directly from one particular cell in the dataset, allows an easy manual back reading of particular data. Although not systematically, a diminution in the number of sudden fluorescent concentration or reversely, of fluorescent local concentration leaving the evanescent field is indeed observable upon nocodazole treatment, in TIRF microscopy image series. Such a result would have been missed without an automatic detection approach. While not really in the scope of this article, a biological hypothesis will be shortly proposed, in the discussion. Other interesting findings extractable from large series of microscopy imaging, is evidently brought by the possibility to analyze quantitatively and with a high degree of confidence, the variability of defined behavior within one particular experimental condition, at the unique cell level. This is illustrated in Figure 8, by the representation of total counts in each single cell.

## Discussion

We here proposed a strategy aimed to automatically select, count, extract and further analyze particular behavior of molecular species involved in intracellular membrane trafficking and revealed by different fluorescence live cells imaging methods. Beside directional transport that most often corresponds to motor driven movement along cytoskeleton of vesicles and tubules, membrane constituents also undergo a number of gathering, sorting, fission, association-dissociation or fusion steps, which can be followed by looking at dedicated fluorescently labeled reporter protein. Series of molecular machines act at each of these steps (lastly reviewed in [34]). Indeed, sorting of lipids and proteins within one particular membrane compartment or membrane domains depends on dynamically regulated complexes, for instance assembled from coat family members, specificity of the coat constituents being defined by both the donor and the acceptor membranes. Reversely, fusion of one particular vesicle with the targeted membrane where their content is delivered to insure its function, leads to the dispersion or the active dissociation of many molecular species. Then, the gathering or the dispersion of many units of one type of them, illustrated here with the help of one selected known reporter protein for the endocytosis-recycling processes, obviously corresponds to dynamic architectures involving the engagement of multiple partners. Knowledge on the biological and biophysical characteristics of some of these compounds at the level of the whole living cell is now made accessible, taking into account the convergence of many technological advances. Yet, in order to sort defined information from apparent stochastic features, improvements in methods allowing a rapid and objective comparison of mass data are required.

One of the major qualitative issues in this respect was to overcome the wide heterogeneity of behaviors undergone by a single structure labeled by a fluorescent protein, including fast and/or long lateral movement and stop and go attitude. This was challenged by a patch-based image processing approach, allowing precise selectivity of fast “emergence” or “fading” of brightly fluorescent signals in a spatially defined location. Results obtained from more than 250 movies, originally obtained from 3D+t or TIRF microscopy, were used in this study. The first conclusion to be drawn from our data validation is the good efficiency of such an approach both for the detection itself and for decision making between motions of the vesicles containing the fluorescent marker and sudden concentrations or disappearances. Other studies dedicated to the semi-automatic detection of similar events have been performed in the past [4], mainly for imaging in TIRF microscopy. One among the added values of the present work is to demonstrate performances compatible with high content screening on large image series, easily usable for different imaging modalities. This looked important to us, since multimodal imaging on the same sample is now more and more available on integrated microscopy systems. As examples of modalities to be targeted, let us quote WF-TIRF or fast confocal-TIRF combined microscopy.

This leads to the important aspect of robustness of our method, for which a number of still limiting considerations must be taken into account. First, given the experiments conducted on synthetic image sequences and reported on Figure 6, it is important that the acquired image sequences lie in the same range of signal-to-noise ratio. This is due to the unavoidable fact that the performances of the detector are function of the signal-to-noise ratio. Second, for raw data validation, acquisitions were performed here using different optics, thoroughly adapted for the respective imaging modalities. Although this was done with the aim to make as best the detection as possible, it makes a direct comparison of the proposed method results between the two imaging methods difficult to appraise. This could be easily solved in the future, by compromising the detector solution and by using the same optical path for both image acquisition techniques. We believe it an important issue to be addressed, since linking temporally 3D+t and 2D+t TIRF microscopy imaging, will give historical information on the fate of membrane structures before and after they move nearby the plasma membrane of the cells. In other words, it will permit eventually to relate these movements with a fast appearance or disappearance, as seen in TIRF microscopy in the case of secretory or recycling proteins.

Despite the needs for expected improvements in hardware solutions, we succeeded to automatically discriminate the different perturbation tests applied to our validation molecular and cellular model, for which some data at the whole cell population had been already achieved. This stands for the over-expression:of the motor mutant form of the Myosin Vb, for which, the proposed approach allowed for the first time to show in TIRF microscopy that Myosin Vb acts primarily as a motor for transport of recycling elements toward the plasma membrane. Moreover, while it was not aimed originally in this study, the described approach allowed to quantify the role of Myosin Vb in the delivery of a transmembrane receptor at the cell surface, in single cell studies.

As a more surprising result still based on preliminary data analysis, the nocodazole effects appear quantitatively antagonist, depending on the microscopy modality. The increased numbers of spot appearance and disappearance in WF could then simply signify that in normal situation (without treatment) microtubules will regulate accessibility of vesicle bearing the Langerin to the plasma membrane. This would also be in agreement with the centro-cellular location of the Langerin imposed by polymerized microtubules and its peripheral redistribution after nocodazole treatment (see Video S3). However, contradictory results obtained in TIRF microscopy, i.e. significant decreased numbers in both fast appearing and vanishing spot events detected, seems to invalidate this vision. Then, an interesting alternative would describe the sudden concentrations and vanishing spots detected, as “packing and unpacking” of the Langerin molecules in and from a particular membrane structure, respectively. This hypothesis would fits with our knowledge of Langerin cell physiology for which such membrane structure, the Birbeck granules, exists. Biogenesis of Birbeck granules depends on both the expression of Langerin itself [3] and of Rab11 membrane domains [2]. That, Langerin under the complex form of Birbeck granules would get access to the plasma membrane, the role of microtubules in this context and the dynamic properties of those specialized structures only identified so far by electronic microscopy approaches in fixed samples, remains to be thoroughly studied and were out of the scope of this paper. However, quantitative assessments of fast dynamic behavior to answer such issues will benefit from the described method.

As further extension of an automatic approach in detecting fast and transient local concentration of proteins within the cell, one may of course envisage the recording of multiple fluorescently labeled entities. A number of simple modifications can be brought to the microscopy systems such as the ones on which data validation was performed. An image splitter or a dual-camera optical part could be integrated allowing stream recording of at least two entities, simultaneously. One could then apply the proposed method for direct co-detection in two fluorescent channels, performing a temporally and spatially defined “co-localization event detection”. Such a molecular pair based approach would be of value to decipher the dynamics of general molecular mechanisms responsible for the different steps in membrane trafficking mentioned above. Alternatively, depending on the relative behavior of the two molecular entities to be considered, one may also use a “space-time cropping” of all detected events in a single channel to examine the occurrence of particular dynamics in the other one, all in an automatic manner.

At least one additional parameter clearly needs to be extracted in the images. While the proposed method actually allows a quite robust detection of appearing or vanishing events in fluorescence, at the moment it lacks the ability to analyze further the fate of those structures, automatically. The automatic tracking over time of any detected sites would be of importance if one wishes to analyze the precise kinetics of fluorescence loss, in order to distinguish between different molecular behaviors such as diffusion, catastrophic or progressive dissociation and further to classify or categorize them at the single cell level.

## Supporting Information

Video S1 Detection result for YFP-Langerin in M10 stable cell lines acquired in wide field microscopy with 200ms of exposure time. The 121 stacks of size 370×404×4 voxels have been processed and are displayed using a maximum intensity projection along the z axis. This video corresponds to Fig 1a. On the right-hand side panel, for each frame, apparition events are displayed in green and vanishing events in red. The uppermost graph on the left represents the intensity inside (orange) and outside the cell (gray) as in Fig. 3. The lowermost graph represents the distribution of the minimal distance between blocks and the corresponding GEV distribution (see Fig. 4).
(0.67 MB MP4)

Video S2 Detection result for YFP-Langerin in M10 stable cell lines acquired in wide field microscopy with 200ms of exposure time. The 121 stacks of size 512×512×4 voxels have been processed and are displayed using a maximum intensity projection along the z axis. This video corresponds to Fig 7a. On the right-hand side panel, for each frame, apparition events are displayed in green and vanishing events in red. The uppermost graph on the left represents the intensity inside (orange) and outside the cell (gray) as in Fig. 3. The lowermost graph represents the distribution of the minimal distance between blocks and the corresponding GEV distribution (see Fig. 4).
(0.68 MB MP4)

Video S3 Detection result for YFP-Langerin in M10 stable cell lines acquired in wide field microscopy with 200ms of exposure time and treated with nocodazole. The 121 stacks of size 382×372×4 voxels have been processed and are displayed using a maximum intensity projection along the z axis. On the right-hand side panel, for each frame, apparition events are displayed in green and vanishing events in red. The uppermost graph on the left represents the intensity inside (orange) and outside the cell (gray) as in Fig. 3. The lowermost graph represents the distribution of the minimal distance between blocks and the corresponding GEV distribution (see Fig. 4).
(0.61 MB MP4)

Video S4 Detection result for YFP-Langerin in M10 stable cell lines acquired in wide field microscopy with 200ms of exposure time and treated with cytochalasin D. The 121 stacks of size 293×335×4 voxels have been processed and are displayed using a maximum intensity projection along the z axis. On the right-hand side panel, for each frame, apparition events are displayed in green and vanishing events in red. The uppermost graph on the left represents the intensity inside (orange) and outside the cell (gray) as in Fig. 3. The lowermost graph represents the distribution of the minimal distance between blocks and the corresponding GEV distribution (see Fig. 4).
(0.69 MB MP4)

Video S5 Detection result for YFP-Langerin in M10 stable cell lines acquired in total internal reflection microscopy with 100ms of exposure time. On the right-hand side panel, for each of the 300 frames of size 358×262 pixels, apparition events are displayed in green and vanishing events in red. The uppermost graph on the left represents the intensity inside (orange) and outside the cell (gray) as in Fig. 3. The lowermost graph represents the distribution of the minimal distance between blocks and the corresponding GEV distribution (see Fig. 4).
(1.55 MB MP4)

Video S6 Detection result for YFP-Langerin in M10 stable cell lines expressing Myosine Vb Tail acquired in total internal reflection microscopy with 100ms of exposure time. On the right panel, for each of the 600 frames of size 229×316 pixels, apparition events are displayed in green and vanishing events in red. The uppermost graph on the left represents the intensity inside (orange) and outside the cell (gray) as in Fig. 3. The lowermost graph represents the distribution of the minimal distance between blocks and the corresponding GEV distribution (see Fig. 4).
(3.10 MB MP4)
